# Combination of a Rapid Diagnostic Assay and Antimicrobial Stewardship Intervention for Gram-Negative Bacteremia

**DOI:** 10.1093/ofid/ofae477

**Published:** 2024-08-22

**Authors:** Julian J Ventres, Michelle H Ting, Diane M Parente, Ralph Rogers, Ashlyn M Norris, Gregorio Benitez, Fadi Shehadeh, April M Bobenchik, Eleftherios Mylonakis, Kimberle C Chapin, Cheston B Cunha

**Affiliations:** Department of Pharmacy, The Miriam Hospital, Providence, Rhode Island, USA; Department of Pharmacy, Banner University Medical Center-Phoenix, Phoenix, Arizona, USA; Department of Pharmacy, The Miriam Hospital, Providence, Rhode Island, USA; Division of Infectious Diseases, Brown University, Warren Alpert Medical School, Providence, Rhode Island, USA; Division of Infectious Diseases, Brown University, Warren Alpert Medical School, Providence, Rhode Island, USA; Division of Infectious Diseases, Brown University, Warren Alpert Medical School, Providence, Rhode Island, USA; Department of Pharmacy, Rhode Island Hospital, Providence, Rhode Island, USA; Division of Infectious Diseases, Brown University, Warren Alpert Medical School, Providence, Rhode Island, USA; Department of Medicine, Houston Methodist Hospital, Houston, Texas, USA; Clinical Pathology Division, Penn State Milton Hershey Medical Center, Hershey, Pennsylvania, USA; Department of Pathology, Penn State College of Medicine, Hershey, Pennsylvania, USA; Department of Medicine, Houston Methodist Hospital, Houston, Texas, USA; Department of Pathology, Brown University, Warren Alpert Medical School, Providence, Rhode Island, USA; Deepull, Barcelona, Spain; Division of Infectious Diseases, Brown University, Warren Alpert Medical School, Providence, Rhode Island, USA; Infectious Disease Division, Rhode Island Hospital and The Miriam Hospital, Providence, Rhode Island, USA

**Keywords:** Accelerate Pheno, antimicrobial stewardship, duration of therapy, gram-negative bacteremia, oral antibiotics

## Abstract

**Background:**

Traditional blood cultures for gram-negative bacteremia can take up to 72 hours or more to return results, prolonging the duration of empiric broad-spectrum intravenous antibiotics. The Accelerate Pheno system provides rapid identification and susceptibilities for blood cultures in gram-negative bacteremia. Current data on its clinical utility are mixed overall, so the system requires further research.

**Methods:**

A multicenter, retrospective quasi-experimental study was conducted comparing the Accelerate Pheno rapid diagnostic system with antimicrobial stewardship intervention and traditional blood cultures alone.

**Results:**

A total of 264 patients with blood cultures with gram-negative bacteria growth were included in the final analysis (102 pre-intervention, 162 post-intervention). The antimicrobial stewardship team made 364 recommendations in 152/162 (93.8%) patients in the post group. Duration of intravenous therapy was shorter (*P* < .001) for the post-intervention group (median, 4.0 days) compared with the pre-intervention group (median, 7.5 days). Hospital length of stay was also shorter (*P* < .001) for the post-intervention group (median, 5.1 days) compared with the pre-intervention group (median, 7.0 days). Readmission rates within 30 days were reduced (*P* = .042) post-intervention (13.0%) compared with pre-intervention (22.6%). In the post-intervention group, a larger proportion of patients were transitioned to oral therapy at any point (126/162, 77.8%) compared with pre-intervention (62/102, 60.8%; *P* < .001).

**Conclusions:**

These results suggest that the Accelerate Pheno system, with active review and intervention by a multidisciplinary antimicrobial stewardship team, is a useful tool in improving both patient-centric and antimicrobial stewardship outcomes.

Effective antimicrobial therapy in patients with bloodstream infections (BSIs) is essential for decreasing morbidity and mortality and helps curb antimicrobial resistance and health care costs [1, 2]. Gram-negative bacteremia specifically is a major cause of severe illness and death, with 7- and 30-day mortality rates of 8% and 15%, respectively [2, 3]. An increasing number of these infections are caused by multidrug-resistant organisms, resulting in poor outcomes and difficulty selecting antimicrobial therapy [4, 5]. Empiric therapy for these infections includes broad-spectrum antibiotics to ensure activity against the most likely causative pathogens and account for drug resistance, with subsequent therapy targeting the specific identified pathogen [6]. Appropriate treatment and narrowing of antimicrobial therapy rely on information from blood cultures, including identification (ID) and antimicrobial susceptibility testing (AST) of the organism. Previous studies have shown that traditional blood cultures can take >24 hours to return an ID following initial bacterial growth and up to 48 hours to return AST results [7]. As a result of the long turnaround time, clinicians are left treating with broad-spectrum antibiotics for extended durations. Unfortunately, both overuse of broad-spectrum antibiotics and longer durations of therapy are associated with increased rates of antimicrobial resistance and *Clostridioides difficile* infection (CDI) [8, 9]. Although antimicrobial stewardship (AMS) intervention after ID and AST results has been shown to be beneficial in multiple ways, including decreasing antibiotic resistance and health care costs, it is still dependent on the time to identification and AST to make appropriate recommendations [1, 10]. As such, improving time to AST results is of high interest, as this would allow for a quicker and greater impact of AMS, cost savings, prevention of adverse events, and optimization of therapy [1].

Several novel technologies have emerged in recent years to address this delay in accurate ID and AST data [10]. One such technology, the Accelerate Pheno system (AXDX; Accelerate Diagnostics, Tucson, AZ, USA), provides ID and AST information for a set panel of common BSI-causing organisms within seven hours of culture positivity [11]. This is performed using a combination of automated fluorescence in situ hybridization to identify organisms and morphokinetic cellular analysis to detect phenotypic characteristics [12]. Once ID and AST have resulted, the AMS team can utilize the information to ensure rapid and appropriate optimization of antimicrobial therapy. Previous studies have already validated the accuracy and speed of AXDX, with concordance with traditional cultures between 96% and 100% for ID and between 91% and 95% for AST [13–15]. More recently, AXDX in conjunction with AMS programs has demonstrated reductions in both duration of broad-spectrum antibiotics and total duration of therapy compared with traditional cultures, both of which are metrics that are extremely relevant to combatting growing antibiotic resistance [16–20].

While recent literature has established many advantages of AXDX, further research is needed on its effects on transitioning from intravenous to oral antibiotics, as well as any effects on length of hospital stay [16–20]. The current study aims to add to this growing body of literature by analyzing the effects of AXDX in conjunction with an AMS program on the duration of intravenous antibiotics in patients with gram-negative bacteremia.

## METHODS

### Study Design

A retrospective, institutional review board–approved, quasi-experimental study was conducted. Data were collected from the electronic medical records of a 719- and a 247-bed hospital, contained within a larger academic health system in Rhode Island, United States, for patients who had blood cultures positive for gram-negative bacteria. In the pre-intervention group, blood was inoculated into Thermo Scientific VersaTREK REDOX1 and REDOX2 bottles (Thermo Scientific, Waltham, MA, USA). Blood culture bottles were then incubated until positive alert, at which point the bottle was gram stained and inoculated to solid media for overnight incubation. Upon overnight incubation, colony identification was performed by Vitek MS (bioMéreieux, Durham, NC, USA), and AST was performed using Vitek 2 AST-GN84 panels (bioMéreieux). ID and AST were then reported via the electronic medical record. There was no routine intervention by AMS on gram-negative bacteremia in the pre-intervention group. In the post-intervention group, blood was inoculated into VersaTREK REDOX1 and REDOX2 bottles, and bottles were incubated until positive alert. Once positive, the bottle was gram stained, inoculated into an AXDX test kit, and inoculated to solid media for overnight incubation. ID and AST from the AXDX test kit were uploaded to the electronic medical record upon completion, and the AMS team was notified via pager. Following overnight incubation, colony identification was performed by Vitek MS, and AST was performed using Vitek 2 AST-GN84 panels; results were uploaded to the electronic medical record. The AMS team reviewed patients for opportunities to optimize, de-escalate, set durations of therapy, and convert antibiotics from intravenous to oral during the hours of 08:00 to 16:30 seven days a week. The AMS team then contacted the primary care team with recommendations for definitive therapy, duration of therapy, and intravenous to oral conversion (if appropriate). For overnight results, the AMS team would review the following morning.

### Population and Inclusion/Exclusion Criteria

**Figure 1.**
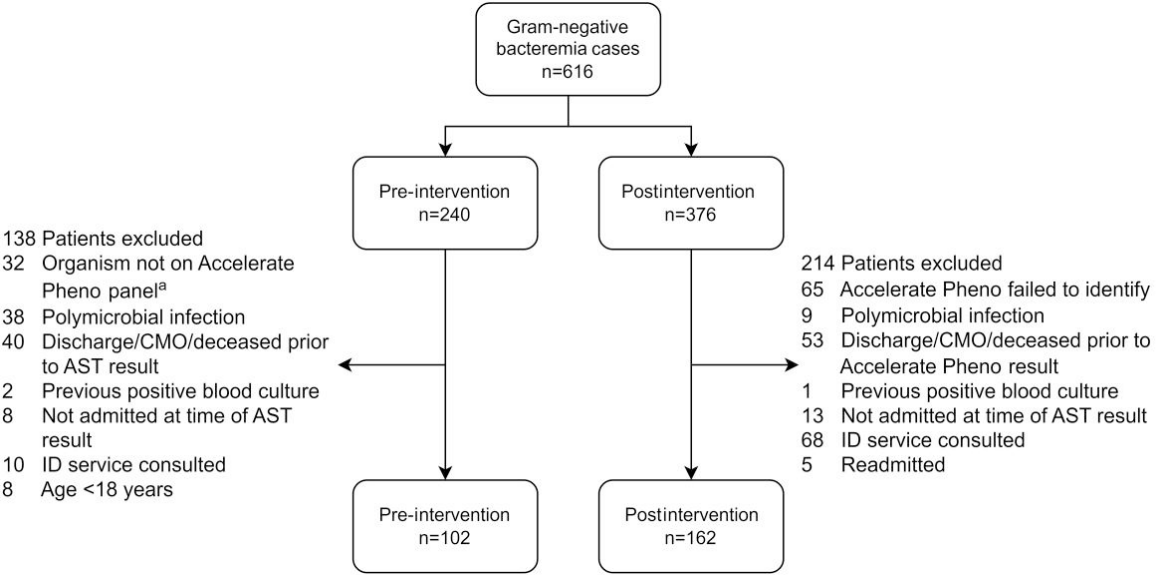
Flowchart representation of included and excluded cases. ^a^Cultures with organisms unable to be identified by the Accelerate Pheno system were excluded from the pre-intervention group. Abbreviations: AST, antimicrobial susceptibility testing; CMO, comfort measures only; ID, infectious disease.

Eligible patients were those admitted to one of the participating sites who had a positive gram stain for a gram-negative organism contained on the panel of the AXDX. Gram-negative organisms tested by the AXDX Test BC Kit include *Klebsiella* spp., *Citrobacter* spp., *Enterobacter* spp., *Proteus* spp., *Escherichia coli, Serratia marcescens, Pseudomonas aeruginosa,* and *Acinetobacter baumannii* [12]. Patients whose blood cultures resulted with gram-negative organisms other than those listed or patients with a prior positive blood culture during the same admission were not included. Patients with polymicrobial BSIs were also not included, as AXDX is unable to reliably report ID and AST for polymicrobial infections [12]. Patients were also excluded if they were <18 years of age, if they were discharged before or within 24 hours of AXDX results, were deceased or made comfort measures only before completion of therapy, or if an infectious disease consult was placed before AXDX results (Figure 1). Lastly, any patients who were re-admitted during the study period only had their first admission included in the study as culture data from the previous admission would provide guidance on empiric therapy for the re-admission.

Data were collected via a retrospective chart review of all patients with positive blood cultures with a gram-negative gram stain from December 1, 2017, to September 1, 2018, for the pre-intervention group, and November 1, 2019, to September 15, 2020, for the post-intervention group. Data points collected included empiric and optimized antimicrobial regimen, time to optimized antibiotic, duration of intravenous and inpatient antibiotics, total duration of antibiotics, and whether any intravenous to oral antibiotic conversions occurred. Safety metrics recorded were readmission within 30 days and development of CDI within 90 days.

### Outcomes

The primary outcome of interest was the duration of intravenous antibiotic therapy, measured in days. Duration of intravenous antibiotics was determined starting on the first day of empiric antibiotics until antibiotics were discontinued or until therapy was transitioned to oral antibiotics, whichever came first. If a patient was discharged on intravenous antibiotics, the prescribed duration at discharge was also included in this count.

Secondary outcomes included time to optimal antimicrobial therapy, defined as the discontinuation of broad-spectrum empiric antibiotics and the initiation of targeted therapy based on the susceptibility pattern of the identified pathogen, counted from the time the blood culture was collected to the time an optimized antimicrobial regimen was ordered, in hours; time to discontinuation, in hours, of anti–methicillin-resistant *Staphylococcus aureus* (anti-MRSA) therapy (vancomycin) and to de-escalation of antipseudomonal beta-lactams (cefepime, piperacillin/tazobactam, and meropenem), counted as the time from start of empiric antibiotics to the time of discontinuation; total duration of antibiotic therapy, in days, including both inpatient and outpatient therapy; rates of conversion from intravenous to oral antibiotics; infection-related length of stay, in days, defined as the time from initial positive culture collection to discharge; total hospital length of stay, in days; incidence of CDI within 90 days of positive culture; and rate of readmission within 30 days of discharge. If antipseudomonal beta-lactams were identified as the optimal treatment agents based on AST results, the cases were excluded from the calculation of time to de-escalation of antipseudomonal beta-lactams.

### Statistical Analysis

Continuous variables were represented as medians with interquartile ranges, and categorical variables were represented as number (%). Both baseline characteristics and study outcomes between intervention groups were evaluated utilizing the Wilcoxon rank-sum test for continuous variables and either the Pearson χ^2^ test for independence or, if appropriate, the Fisher exact test for categorical variables.

Significant tests for baseline characteristics were further analyzed in post hoc analyses in which critical *P* values were adjusted for multiple comparisons using Bonferroni's correction. Analyses were performed using Stata software, version 17.0 (StataCorp). Statistical significance was set at a critical *P* value of ≤.05.

## RESULTS

A total of 616 blood cultures positive for gram-negative rod-shaped organisms were collected during the study periods. After exclusions, 264 patients were included for analysis, with 102 patients in the pre-intervention group and 162 patients in the post-intervention group. Patient sex assigned at birth, age, and Charlson comorbidity index were similar between the 2 groups (Table 1). The majority of infections were genitourinary in origin (66.3%), followed by gastrointestinal (20.1%). The most common organisms identified were *E. coli* (61.0%), *Klebsiella* spp. (21.2%), and *Proteus* spp. (6.8%). Although the most common causative microbes and sources of infection were similar in both groups, significant differences were observed in the overall distribution of causative microbe and source of infection between groups (Supplementary Tables 1 and 2).

**Table 1.** Baseline Characteristics

Characteristic	Pre-intervention (n = 102)	Post-intervention (n = 162)	Total (n = 264)	*P* Value
Age, y	71.5 (57–81)	72 (62–81)	72 (59.5–81)	.441
Male sex	51 (50)	79 (48.8)	130 (49.2)	.845
Charlson Comorbidity Score	1 (0–3)	1 (0–3)	1 (0–3)	.314
Presumed source	…	…	…	**<.001**
Genitourinary	54 (52.94)	121 (74.69)	175 (66.29)	
Gastrointestinal	26 (25.49)	27 (16.67)	53 (20.08)	
Bone/joint	4 (3.92)	1 (0.62)	5 (1.89)	
SSTI	5 (4.90)	1 (0.62)	6 (2.27)	
CLABSI	10 (9.80)	2 (1.23)	12 (4.55)	
Other^a^	3 (2.94)	10 (6.17)	13 (4.92)	
Isolated bacteria	…	…	…	**<.001**
*Escherichia coli*	57 (55.88)	104 (64.20)	161 (60.98)	
*Klebsiella* species	25 (24.51)	31 (19.14)	56 (21.21)	
*Pseudomonas aeruginosa*	0 (0.00)	9 (5.56)	9 (3.41)	
*Enterobacter* species	3 (2.94)	7 (4.32)	10 (3.79)	
*Proteus* species	7 (6.86)	11 (6.79)	18 (6.82)	
Other^b^	10 (9.80)	0 (0.00)	10 (3.79)	

Age and Charlson Comorbidity Score are reported as median (IQR). Sex, presumed source, and isolated bacteria are reported as number (%). *P* values were calculated using the Wilcoxon rank-sum test for continuous variables and either the Pearson χ^2^ test or the Fisher exact test for categorical variables. Statistically significant values are in bold (*P* ≤ .05).

Abbreviations: CLABSI, central line–associated bloodstream infection; IQR, interquartile range; SSTI, skin and soft tissue infection.

^a^Pneumonia, neutropenic fever, graft infection, spontaneous bacterial peritonitis, unknown source.

^b^ *Citrobacter* species, *Acinetobacter baumannii, Serratia marcescens*.

In the post-intervention group, the AMS team made 369 recommendations on 152/162 (93.8%) patients. Of the 369 recommendations made, 296 interventions (80.2%) were accepted (Table 2). The most common interventions were duration of therapy (29.7%), intravenous to oral conversion (28.6%), and de-escalation of therapy (28.6%).

**Table 2.** Recommendations Made by the Antimicrobial Stewardship Team in the Post-intervention Group

Intervention Type	Occurrences
IV to PO	106 (28.72)
Duration	110 (29.81)
De-escalation	104 (28.18)
Escalation/added agent	13 (3.52)
Labs/imaging	9 (2.44)
Infectious disease consult	17 (4.61)
Other	10 (2.71)

Reported as number (%).

Abbreviations: IV, intravenous; PO, per os.

Duration of intravenous therapy was shorter (*P* < .001) in the post-intervention group (median, 4.0 days) compared with the pre-intervention group (median, 7.5 days) (Table 3). This was paired with a higher rate of conversion from intravenous to oral therapy, with 126/162 (77.8%) converted in the post-intervention group and 62/102 (60.8%) converted in the pre-intervention group (*P* = .003). Total duration of antibiotic therapy was also shorter (*P* < .001) for the post-intervention group (median, 13 days) compared with the pre-intervention group (median, 15 days).

**Table 3.** Measured Outcomes

Outcome	Pre-intervention (n = 102)	Post-intervention (n = 162)	*P* Value
LOS			
Hospital LOS, d	7.01 (4.93–10.96)	5.09 (3.45–8.37)	**<.001**
Infection-related LOS, d	5.95 (4.04–9.72)	3.86 (2.62–6.98)	**<.001**
Optimal therapy			
Duration of IV therapy, d	7.5 (5.0–15.0)	4 (3.0–7.0)	**<.001**
Total duration of antibiotic therapy, d	15 (14–17)	13 (8–15)	**<.001**
Time to optimization, h	64.20 (52.03–90.78)	20.20 (12.43–30.95)	**<.001**
Time to discontinuation of anti-MRSA therapy, d	(n = 65) 2.2 (0.99–3.28)	(n = 77) 1.32 (0.83–1.78)	**.002**
Time to discontinuation of antipseudomonal beta-lactam, d	(n = 84) 3.44 (2.68–4.70)	(n = 122) 1.50 (1.00–2.15)	**<.001**
Conversion from IV to oral therapy	62 (60.8)	126 (77.8)	**.003**
Safety outcomes			
Rates of CDI within 90 d	7 (6.9)	5 (3.1)	.224
Rates of readmission within 30 d	23 (22.6)	21 (13)	**.042**

Data are reported as either No. (%) or median (IQR). *P* values were calculated using the Wilcoxon rank-sum test for continuous variables and either the Pearson χ^2^ test or the Fisher exact test for categorical variables. Statistically significant values are in bold (*P* ≤ .05). Abbreviations: CDI, *Clostridioides difficile* infection; IQR, interquartile range; IV, intravenous; LOS, length of stay; MRSA, methicillin-resistant *Staphylococcus aureus*.

Time to optimal antimicrobial therapy was shorter (*P* < .001) for the post-intervention group (median, 20.2 hours) compared with the pre-intervention group (median, 64.2 hours). In patients receiving anti-MRSA or antipseudomonal beta-lactams, time to appropriate discontinuation of these agents was also reduced in the post-intervention group. Time to discontinuation of anti-MRSA therapy was shorter (*P* = .002) for the post-intervention group (median, 1.3 days) compared with the pre-intervention group (median, 2.2 days). Additionally, time to discontinuation of antipseudomonal beta-lactams was shorter (*P* < .001) for the post-intervention group (median, 1.5 days) compared with the pre-intervention group (median, 3.4 days).

Length of stay was shorter (*P* < .001) for the post-intervention group (median, 5.1 days) compared with the pre-intervention group (median, 7.0 days) (Table 3). Similarly, median infection-related length of stay was also shorter (*P* < .001) in the post-intervention group (median, 3.9 days) compared with the pre-intervention group (median, 6.0 days).

Regarding safety metrics, there was a numerically decreased but nonsignificant reduction observed between the pre- and post-intervention groups in rates of CDI within 90 days of positive culture (6.9% vs 3.1%, respectively; *P* = .224) (Table 3). Readmission rates within 30 days were reduced in the post-intervention compared with pre-intervention group (13.0% vs 22.6%; *P* = .042).

## DISCUSSION

The present study's goal was to evaluate the clinical impact of a rapid identification and phenotypic AST assay with intervention by an AMS team. Implementation of this process was associated with significant improvements in nearly all measured AMS metrics. More strikingly, we also found reductions in duration of hospitalization and readmission rates in the post-intervention group.

A significant reduction in the duration of intravenous antibiotic therapy was observed after the addition of AXDX with intervention by an AMS team. Intravenous therapy is primarily used as initial therapy in the inpatient setting, to quickly acquire adequate systemic levels of antibiotics, or in patients with impaired oral absorption. While these benefits are important in initial management of more severe infections, intravenous therapy also carries risks, including catheter-related infections, thromboembolisms, and extravasation. Early conversion to oral therapy in gram-negative bacteremia is well established to be safe and effective in clinically stable and improving patients with functioning gastrointestinal tracts [21, 22]. Additionally, other studies have found that conversion to oral medications confers significant cost-savings to the hospital system [23, 24]. The observed reduction in duration of intravenous therapy among our cohort was likely a result of the combination of active intervention by the AMS team alongside access to quicker identification and AST results via the rapid diagnostic platform implemented here.

Unique to this study, we observed a significant increase in the proportion of patients transitioned to oral antibiotics. Previous studies have either not reported this metric or have not seen a difference in conversion rate [16]. The major driver of this outcome is likely the added review and intervention by the AMS team. While Walsh et al. did not see a significant difference in their study, they included active AMS intervention both before and after the implementation of AXDX, and as such AMS was likely already increasing intravenous-to-oral conversion in the pre-intervention group [16]. In contrast, our study only included AMS intervention in the post-implementation group, and thus it seems likely that this intervention may have resulted in the observed increased rate of therapy completion with oral antibiotics. Of note, our population in both groups was generally healthy at baseline, with a median Charlson Comorbidity Index of 1, indicating a relatively low occurrence of comorbidities that may complicate conversion to oral therapy. While outpatient parenteral antimicrobial therapy (OPAT) is sometimes necessary, it carries several disadvantages compared with oral antibiotics. Previous literature has shown that OPAT imposes significant cost burdens on patients, as well as reductions in quality of life, compared with oral therapy [25]. Up to 9% of patients receiving OPAT will experience a complication with vascular access during their therapy [26]. Additionally, OPAT continues to be a challenging option for vulnerable populations, such as people experiencing homelessness and people who inject drugs, due to concerns with the safe administration of parenteral therapy outside of the hospital [27]. Increasing rates of completion with oral therapy work to reduce OPAT-associated complications and increase accessibility to effective antimicrobial therapy within these populations.

Compared with the pre-intervention cohort, the duration of both antipseudomonal beta-lactams and anti-MRSA therapy was reduced. The quicker time to de-escalate from antipseudomonal beta-lactams can likely be credited to the faster identification of the causative microbe, as one would be unable to de-escalate this based on the results of gram stain alone. As AXDX is only run on gram-negative gram stains at the included institutions, the reduced duration of anti-MRSA therapy in the post-intervention group was unexpected based on the assumption that clinicians would discontinue anti-MRSA antibiotics as soon as the gram stain resulted as gram-negative, and thus this reduction may have been driven primarily by the AMS intervention as opposed to the rapid diagnostics.

We observed a reduction in both infection-related length of stay and total hospital length of stay following the addition of this diagnostic test in conjunction with active intervention by our AMS team. When evaluating length of stay, previous studies have shown mixed results overall [12]. One study observed no difference in length of stay in a pediatric population, while another saw a significant reduction in length of stay in patients with gram-negative BSIs [28, 29]. Bhalodi et al. did not observe any difference when looking at gram-positive and gram-negative BSIs but did note a significant reduction when only looking at gram-negative BSIs [30]. Lastly, Dare et al. showed a significant decrease in length of stay for gram-negative BSIs with rapid diagnostics plus AMS with or without real-time notification compared with traditional testing but observed no difference between AMS intervention with and without real-time notification [20]. Of the above-described studies, those that showed a reduction in length of stay incorporated active intervention by an AMS team as part of their study, whereas those without significant length of stay reductions did not. Our study further supports that effective utilization of the rapid diagnostics provided by AXDX, when used alongside an AMS team, significantly reduces hospital length of stay for gram-negative BSIs. The differences seen in our study compared with previous studies, both in methodology and results, underpin the importance of the AMS team component when implementing rapid diagnostic testing to achieve the greatest benefit.

There are several limitations present within this study. The pre- and post-intervention design is at risk of confounding due to practice changes over time, through guideline and literature updates as well as institutional changes. Both source of infection and causative microbe were significantly different between the cohorts in the study; however, the most common sources and microbes were the same. Post hoc analyses revealed that the differences in source were driven by the number of genitourinary and central line–associated BSIs between the two groups, while differences in bacterial species were driven by the presence of ten infections by *Citrobacter* spp., *Serratia marcescens,* and *Acinetobacter baumannii* in the pre-intervention group, with none of these organisms occurring in the post-intervention group (Supplementary Tables 1 and 2). The larger number of nongenitourinary infections may have contributed to worse outcomes in the pre-intervention group, as urosepsis has been shown to have better outcomes than other sources of infection [31]. Only readmissions to facilities within the hospital network were able to be captured, and thus a portion of readmissions may have been missed. A large number of patients were excluded; however, the applied exclusion criteria were necessary for the comparison of AXDX with AMS vs traditional cultures alone. Exclusion of polymicrobial/off-panel organisms, for example, was necessary as AXDX is unable to reliably identify organisms in these scenarios, and thus when a gram stain suggested multiple morphologies the culture was not run on AXDX. Similarly, as we were looking at AMS combined with AXDX, infectious diseases consults were excluded as AMS would no longer be recommending therapy at that point. Lastly, the AMS team reviewed results only between the hours of 08:00 and 16:30, which may have limited the benefit of the rapid turnaround time of AXDX. Real-time review of results 24 hours per day may provide improved results; however, this is unlikely to be feasible due to staffing limitations at most institutions.

While this study demonstrated improvement in several measurable clinical outcomes associated with the use of AXDX, several metrics remain to be evaluated. Although it can be assumed that reducing length of hospital stay is likely to provide significant economic benefits, the degree of this benefit would need to be weighed against the costs of AXDX and further evaluated in follow-up studies to fully quantify. A significant reduction in readmission rate in the post-intervention group compared with the pre-intervention group was observed as well; however, our study sample size may not have been large enough to capture the true event rate between groups.

## CONCLUSIONS

Rapid diagnostic tests in conjunction with intervention by an AMS team for gram-negative BSIs offer many advantages over traditional cultures alone. This study supports previously observed conclusions of decreased time to optimal therapy, reduced length of stay, and reduced duration of antibiotic therapy. In addition to these findings, this study also showed a reduction in duration of intravenous therapy, alongside an increased rate of conversion to oral antibiotics compared with traditional cultures alone. These differences likely reduce costs, increase patient quality of life, and improve access to care. Overall, these results suggest a definite clinical utility associated with integration of AXDX in conjunction with review by an AMS team as compared with the use of a traditional automated blood culture system without AMS intervention.

## Supplementary Data

Supplementary materials are available at *Open Forum Infectious Diseases* online. Consisting of data provided by the authors to benefit the reader, the posted materials are not copyedited and are the sole responsibility of the authors, so questions or comments should be addressed to the corresponding author.

## Supplementary Material

ofae477_Supplementary_Data

## References

[1] Doron S, Davidson LE. Antimicrobial stewardship. Mayo Clin Proc 2011; 86:1113–23. 22033257 10.4065/mcp.2011.0358 PMC3203003

[2] Fitzpatrick J, Biswas J, Edgeworth J, et al Gram-negative bacteremia; a multi-centre prospective evaluation of empiric antibiotic therapy and outcome in English acute hospitals. Clin Microbiol Infect 2016; 22:244–51. 26577143 10.1016/j.cmi.2015.10.034

[3] Angus DC, van der Poll T. Severe sepsis and septic shock. N Engl J Med 2013; 369:840–51. Erratum in: N Engl J Med 2013; 369:2069. 23984731 10.1056/NEJMra1208623

[4] Leal H, Azevedo J, Silva G, et al Bloodstream infections caused by multidrug resistant gram-negative bacteria: epidemiological, clinical and microbiological features. BMC Infect Dis 2019; 19:609. 31296179 10.1186/s12879-019-4265-z PMC6624930

[5] Antimicrobial Resistance Collaborators . Global burden of bacterial antimicrobial resistance in 2019: a systematic analysis. Lancet 2022; 399:629–55. 35065702 10.1016/S0140-6736(21)02724-0 PMC8841637

[6] Terreni M, Taccani M, Pregnolato M. New antibiotics for multidrug-resistant bacterial strains: latest research developments and future perspectives. Molecules 2021; 26:2671. 34063264 10.3390/molecules26092671 PMC8125338

[7] Tabak YP, Vankeepuram L, Ye G, Jeffers K, Gupta V, Murray PR. Blood culture turnaround time in U.S. acute care hospitals and implications for laboratory process optimization. J Clin Microbiol 2018; 56:e00500–18. 30135230 10.1128/JCM.00500-18 PMC6258864

[8] Eze P, Balsells E, Kyaw MH, Nair H. Risk factors for *Clostridium difficile* infections—an overview of the evidence base and challenges in data synthesis. J Glob Health 2017; 7:010417. 28607673 10.7189/jogh.07.010417 PMC5460399

[9] Cižman M, Plankar Srovin T. Antibiotic consumption and resistance of gram-negative pathogens (collateral damage). GMS Infect Dis 2018; 6:Doc05. 30671336 10.3205/id000040 PMC6301726

[10] Timbrook TT, Hurst JM, Bosso JA. Impact of an antimicrobial stewardship program on antimicrobial utilization, bacterial susceptibilities, and financial expenditures at an academic medical center. Hosp Pharm 2016; 51:703–11. 27803499 10.1310/hpj5109-703 PMC5080988

[11] Trotter A, Aydin A, Strinden M, et al Recent and emerging technologies for the rapid diagnosis of infection and antimicrobial resistance. Curr Opin Microbiol 2019; 51:39–45. 31077935 10.1016/j.mib.2019.03.001

[12] Accelerate Diagnostics, Inc. Accelerate Pheno® system. Available at: https://acceleratediagnostics.com/products/accelerate-pheno-system/. Accessed 19 August 2022.

[13] Cenci E, Paggi R, Socio GV, et al Accelerate Pheno® blood culture detection system: a literature review. Future Microbiol 2020; 15:1595–605. 33215528 10.2217/fmb-2020-0177

[14] Descours G, Desmurs L, Hoang TLT, et al Evaluation of the Accelerate Pheno® system for rapid identification and antimicrobial f testing of gram-negative bacteria in bloodstream infections. Eur J Clin Microbiol Infect Dis 2018; 37:1573–83. 29808350 10.1007/s10096-018-3287-6

[15] Pantel A, Monier J, Lavigne JP. Performance of the Accelerate Pheno® system for identification and antimicrobial susceptibility testing of a panel of multidrug-resistant gram-negative bacilli directly from positive blood cultures. J Antimicrob Chemother 2018; 73:1546–52. 29474660 10.1093/jac/dky032

[16] Walsh TL, Bremmer DN, Moffa MA, et al Impact of an antimicrobial stewardship program-bundled initiative utilizing Accelerate Pheno® system in the management of patients with aerobic gram-negative bacilli bacteremia. Infection 2021; 49:511–9. 33528813 10.1007/s15010-021-01581-1 PMC8159835

[17] Sheth S, Miller M, Prouse AB, Baker S. Pharmacist-driven implementation of fast identification and antimicrobial susceptibility testing improves outcomes for patients with gram-negative bacteremia and candidemia. Antimicrob Agents Chemother 2020; 64:e00578–20. 32601164 10.1128/AAC.00578-20 PMC7449197

[18] Babowicz F, LaPlante R, Mitchell C, et al Impact of Accelerate Pheno® and BacT/alert virtuo on clinical processes and outcomes in patients with sepsis and concurrent gram-negative bacteremia. Antimicrob Agents Chemother 2021; 65:e02364–20. 33753337 10.1128/AAC.02364-20 PMC8315910

[19] Banerjee R, Komarow L, Virk A, et al Randomized trial evaluating clinical impact of RAPid IDentification and susceptibility testing for gram-negative bacteremia: RAPIDS-GN. Clin Infect Dis 2021; 73:e39–46. 32374822 10.1093/cid/ciaa528 PMC8246790

[20] Dare RK, Lusardi K, Pearson C, et al Clinical impact of Accelerate Pheno® rapid blood culture detection system in bacteremic patients. Clin Infect Dis 2021; 73:e4616–26. 32463864 10.1093/cid/ciaa649

[21] Al-Hasan MN, Rac H. Transition from intravenous to oral antimicrobial therapy in patients with uncomplicated and complicated bloodstream infections. Clin Microbiol Infect 2020; 26:299–306. 31128289 10.1016/j.cmi.2019.05.012

[22] Tamma PD, Conley AT, Cosgrove SE, et al Association of 30–day mortality with oral step-down vs continued intravenous therapy in patients hospitalized with Enterobacteriaceae bacteremia. JAMA Intern Med 2019; 179:316–23. Erratum in: JAMA Intern Med 2019; 179:1607. 30667477 10.1001/jamainternmed.2018.6226 PMC6439703

[23] Lau BD, Pinto BL, Thiemann DR, Lehmann CU. Budget impact analysis of conversion from intravenous to oral medication when clinically eligible for oral intake. Clin Ther 2011; 33:1792–6. 22001356 10.1016/j.clinthera.2011.09.030

[24] Mok M, Kinkade A, Tung A, Tejani AM. Identification of patients eligible for IV-to-PO conversion: a cost-minimization study. Can J Hosp Pharm 2016; 69:301–5. 27621490 10.4212/cjhp.v69i4.1584 PMC5008426

[25] Krah NM, Bardsley T, Nelson R, et al Economic burden of home antimicrobial therapy: OPAT versus oral therapy. Hosp Pediatr 2019; 9:234–40. 30885919 10.1542/hpeds.2018-0193 PMC6434972

[26] Shrestha NK, Shrestha J, Everett A, et al Vascular access complications during outpatient parenteral antimicrobial therapy at home: a retrospective cohort study. J Antimicrob Chemother 2016; 71:506–12. 26510718 10.1093/jac/dkv344

[27] Beieler A, Magaret A, Zhou Y, Schleyer A, Wald A, Dhanireddy S. Outpatient parenteral antimicrobial therapy in vulnerable populations—people who inject drugs and the homeless. J Hosp Med 2019; 14:105–9. 30785418 10.12788/jhm.3138 PMC6996559

[28] Truong TT, Mongkolrattanothai K, Flores II, Dien Bard J. Evaluation of the performance and clinical impact of a rapid phenotypic susceptibility testing method directly from positive blood culture at a pediatric hospital. J Clin Microbiol 2022; 60:e0012222. 35852363 10.1128/jcm.00122-22 PMC9383260

[29] Roth F, Leedahl ND, Leedahl DD, Guerrero DM. Clinical and financial impact of rapid antimicrobial susceptibility testing in blood cultures. Antibiotics (Basel) 2022; 11:122. 35203725 10.3390/antibiotics11020122 PMC8868382

[30] Bhalodi AA, MacVane SH, Ford B, et al Real-World impact of the Accelerate Pheno® test BC kit on patients with bloodstream infections in the improving outcomes and antimicrobial stewardship study: a quasiexperimental multicenter study. Clin Infect Dis 2022; 75:269–77. 34718456 10.1093/cid/ciab921 PMC9410719

[31] Leligdowicz A, Dodek PM, Norena M, Wong H, Kumar A. Co-operative antimicrobial therapy of septic shock database research group. Association between source of infection and hospital mortality in patients who have septic shock. Am J Respir Crit Care Med 2014; 189:1204–13. 24635548 10.1164/rccm.201310-1875OC

