# Supplementary material for: Combination of a Rapid Diagnostic Assay and Antimicrobial Stewardship Intervention for Gram-Negative Bacteremia
Source: Open Forum Infect Dis. 2024 Aug 22;11(9):ofae477. doi: 10.1093/ofid/ofae477 (PMC11389609; doi:10.1093/ofid/ofae477)
Supplement: ofae477_Supplementary_Data [file ofae477_supplementary_data.docx]

**Supplementary Appendix**

**Supplement to:** Combination of a Rapid Diagnostic Assay and Antimicrobial Stewardship Intervention on Gram-negative Bacteremia

**Contents:**

Table S1: Post-hoc analysis of infectious sources between intervention groups

Table S2: Post-hoc analysis of causative pathogens between intervention groups

Table S1: Post-hoc analysis of infectious sources between intervention groups

|  | Genitourinary | Gastrointestinal | Bone/joint | SSTI | CLABSI | Other^a^ |
| --- | --- | --- | --- | --- | --- | --- |
| Genitourinary |  | 0.015 | 0.038 | 0.015 | **< 0.001** | 0.757 |
| Gastrointestinal | 0.015 |  | 0.354 | 0.198 | 0.051 | 0.123 |
| Bone/joint | 0.038 | 0.354 |  | 1.000 | 1.000 | 0.047 |
| SSTI | 0.015 | 0.198 | 1.000 |  | 1.000 | 0.041 |
| CLABSI | **< 0.001** | 0.051 | 1.000 | 1.000 |  | 0.005 |
| Other^a^ | 0.757 | 0.123 | 0.047 | 0.041 | 0.005 |  |

Critical p-value adjusted for multiple comparisons using Bonferroni correction. Statistical significance set at < 0.003. Bold-faced text indicates statistical significance. Each cell presents a p-value from either Pearson χ^2^ test for independence or, if appropriate, Fisher’s exact test comparing the association between respective infectious sources and intervention group status. Post-hoc analysis revealed that genitourinary and CLABSI sources were statistically different between intervention groups. Specifically, 54/64 (84.38%) of patients in the pre-intervention group and 121/123 (98.37%) of patients in the post-intervention group had an infection of genitourinary origin compared with a CLABSI. ^a^Pneumonia, neutropenic fever, graft infection, spontaneous bacterial peritonitis, unknown source*.* Abbreviations: SSTI, skin and soft tissue infection; CLABSI, central line-associated bloodstream infection.

Table S2: Post-hoc analysis of causative pathogens between intervention groups

|  | *Escherichia coli* | *Klebsiella* species | *Pseudomonas aeruginosa* | *Enterobacter* species | *Proteus* species | Other^a^ |
| --- | --- | --- | --- | --- | --- | --- |
| *Escherichia coli* |  | 0.219 | 0.030 | 1.000 | 0.799 | **< 0.001** |
| *Klebsiella* species | 0.219 |  | 0.010 | 0.498 | 0.787 | **0.001** |
| *Pseudomonas aeruginosa* | 0.030 | 0.010 |  | 0.211 | 0.059 | **< 0.001** |
| *Enterobacter* species | 1.000 | 0.498 | 0.211 |  | 0.703 | **0.003** |
| *Proteus* species | 0.799 | 0.787 | 0.059 | 0.703 |  | **0.002** |
| Other^a^ | **< 0.001** | **0.001** | **< 0.001** | **0.003** | **0.002** |  |

Critical p-value adjusted for multiple comparisons using Bonferroni correction. Statistical significance set at < 0.003. Bold-faced text indicates statistical significance. Each cell presents a p-value from either Pearson χ^2^ test for independence or, if appropriate, Fisher’s exact test comparing the association between respective causative pathogens and intervention group status. Post-hoc analysis revealed that the difference in causative pathogen between intervention groups was driven by infection by *Citrobacter* species, *Acinetobacter* *baumannii, or Serratia marcescens* only occurring in patients from the pre-intervention group. ^a^*Citrobacter* species, *Acinetobacter baumannii*, and *Serratia marcescens*
